# Supplementary material for: From sequence to enzyme mechanism using multi-label machine learning
Source: BMC Bioinformatics. 2014 May 19;15:150. doi: 10.1186/1471-2105-15-150 (PMC4229970; doi:10.1186/1471-2105-15-150)
Supplement: Additional file 2 — Java code of ml2db. Additional file ml2db_code.tar.gz contains the Java source code to run the multi-label machine learning experiments and save the results to database. The code’s Javadoc is included. [file 1471-2105-15-150-S2.zip › additional file 2/ml2db/utils/doc/uk/ac/standrews/utils/main/Utils.html]

Utils


JavaScript is disabled on your browser.


- Overview
- Package
- Class
- Use
- Tree
- Deprecated
- Index
- Help

- Prev Class
- Next Class

- Frames
- No Frames

- All Classes

- Summary:
- Nested |
- Field |
- Constr |
- Method

- Detail:
- Field |
- Constr |
- Method


uk.ac.standrews.utils.main

## Class Utils

- java.lang.Object
- - uk.ac.standrews.utils.main.Utils

- ---

    

  ```
  public class Utils
  extends java.lang.Object
  ```

  Various utilities to manipulate vectors of numbers, get a timestamp etc.

  Version:
  :   24-Mar-2007

  Author:
  :   Luna De Ferrari (luna.deferrari at ed.ac.uk)

- - ### Constructor Summary

    Constructors

    Constructor and Description

    `Utils()`
  - ### Method Summary

    Methods

    | Modifier and Type | Method and Description |
    | --- | --- |
    | `static double` | `calcAverage(double[] values)` Calculates the average of the numbers in an array (from web http://forum.java.sun.com/, author JosAH) |
    | `static double` | `calcStandardDeviation(double[] values)` Calculates the standard deviation of the elements in an array as root of the variance = [sum( x(i) - mean)^2 )] / (n-1), where x(i) is the ith element, m the mean, n the number of elements |
    | `static java.util.TreeSet<java.lang.Double>` | `getDoubleIntervals(java.lang.Double min, java.lang.Double max, java.lang.Double percentOfIncrement)` |
    | `static boolean` | `getFlag(java.lang.String opt, java.lang.String[] options)` |
    | `static java.util.TreeSet` | `getIncrements(java.lang.Object min, java.lang.Object max, java.lang.Double percentageOfIncrement)` Returns a vector of numbers, starting from a minimum value, with incremental increases until the maximum value. |
    | `static java.util.TreeSet<java.lang.Integer>` | `getIncrementsFromRange(java.lang.Integer range, java.lang.Double percentageOfIncrement)` Returns a set of increments. |
    | `static java.util.TreeSet<java.lang.Integer>` | `getIntegerIntervals(java.lang.Integer min, java.lang.Integer max, java.lang.Double percentageOfIncrement)` Returns a vector of numbers, starting from a minimum value, with incremental increases until the maximum value. |
    | `static void` | `getJavaSystemProperties()` Gets the Java system properties |
    | `static java.lang.String` | `getOption(java.lang.String opt, java.lang.String[] options)` getOption(String opt, String[] options) gets an option value out of a list of options |
    | `static java.util.TreeSet<java.lang.String>` | `getSimpleSerie(java.lang.Double min, java.lang.Double increment, int iterations)` Gets a series of doubles from a min incrementing by a given increment for a number of iterations |
    | `static java.lang.Boolean` | `implementsInterface(java.lang.Object object, java.lang.Class interf)` |
    | `static void` | `main(java.lang.String[] argv)` Main method for testing |
    | `static java.util.Properties` | `readLocalProperties(java.lang.String resourceName)` Reads properties that inherit from three locations. |
    | `static java.util.Properties` | `readProperties(java.lang.String propertiesFilePath)` Reads properties Code adapted from Weka: http://www.cs.waikato.ac.nz/ml/weka/ Ian H. |
    | `static double` | `roundDouble(double targetDouble, int decimalPlace)` Rounds a number to just n decimal digits |
    | `static java.util.TreeSet<java.lang.String>` | `setFromObjectsToString(java.util.TreeSet<java.lang.Object> set)` |

    - ### Methods inherited from class java.lang.Object

      `equals, getClass, hashCode, notify, notifyAll, toString, wait, wait, wait`

- - ### Constructor Detail


    - #### Utils

      ```
      public Utils()
      ```
  - ### Method Detail


    - #### calcAverage

      ```
      public static double calcAverage(double[] values)
      ```

      Calculates the average of the numbers in an array (from web
      http://forum.java.sun.com/, author JosAH)

      Returns:
      :   average of the numbers (rounded to 2 decimal digits)


    - #### calcStandardDeviation

      ```
      public static double calcStandardDeviation(double[] values)
      ```

      Calculates the standard deviation of the elements in an array as root of
      the variance = [sum( x(i) - mean)^2 )] / (n-1), where x(i) is the ith
      element, m the mean, n the number of elements

      Returns:
      :   standard deviation


    - #### getDoubleIntervals

      ```
      public static java.util.TreeSet<java.lang.Double> getDoubleIntervals(java.lang.Double min,
                                                           java.lang.Double max,
                                                           java.lang.Double percentOfIncrement)
      ```


    - #### getFlag

      ```
      public static boolean getFlag(java.lang.String opt,
                    java.lang.String[] options)
      ```


    - #### getIncrements

      ```
      public static java.util.TreeSet getIncrements(java.lang.Object min,
                                    java.lang.Object max,
                                    java.lang.Double percentageOfIncrement)
      ```

      Returns a vector of numbers, starting from a minimum value, with
      incremental increases until the maximum value. Example: for min 2 and max
      12, percentage of increment 0.2 > [2, 4, 6, 8, 10, 12]

      Parameters:
      :   `min` - the minimum number value
      :   `max` - the maximum number value
      :   `percentageOfIncrement` - the increment as percentage of (maximum - minimum) value (the
          actual increment value has to be >= 1 !)

      Returns:
      :   a vector of numbers, starting from a minimum value, with
          incremental increases until the maximum value. Returns null if
          the increment is 0.


    - #### getIncrementsFromRange

      ```
      public static java.util.TreeSet<java.lang.Integer> getIncrementsFromRange(java.lang.Integer range,
                                                                java.lang.Double percentageOfIncrement)
      ```

      Returns a set of increments. Example: for range 10, percentage of
      increment 0.2 > [0, 2, 4, 6, 8, 10]

      Parameters:
      :   `range` -
      :   `percentageOfIncrement` -

      Returns:
      :   a set of increments


    - #### getIntegerIntervals

      ```
      public static java.util.TreeSet<java.lang.Integer> getIntegerIntervals(java.lang.Integer min,
                                                             java.lang.Integer max,
                                                             java.lang.Double percentageOfIncrement)
      ```

      Returns a vector of numbers, starting from a minimum value, with
      incremental increases until the maximum value.

      Parameters:
      :   `min` - the minimum number value
      :   `max` - the maximum number value
      :   `percentageOfIncrement` - the increment as percentage of (maximum - minimum) value

      Returns:
      :   a vector of numbers, starting from a minimum value, with
          incremental increases until the maximum value. Returns null if
          the increment is 0.


    - #### getJavaSystemProperties

      ```
      public static void getJavaSystemProperties()
      ```

      Gets the Java system properties


    - #### getOption

      ```
      public static java.lang.String getOption(java.lang.String opt,
                               java.lang.String[] options)
      ```

      getOption(String opt, String[] options) gets an option value out of a
      list of options

      Parameters:
      :   `opt` - the option that needs to be extracted (e.g. -x)
      :   `options` - list of options (e.g. -x 10 -y name)

      Returns:
      :   the value for the option selected (e.g. 10)


    - #### getSimpleSerie

      ```
      public static java.util.TreeSet<java.lang.String> getSimpleSerie(java.lang.Double min,
                                                       java.lang.Double increment,
                                                       int iterations)
      ```

      Gets a series of doubles from a min incrementing by a given increment for
      a number of iterations

      Parameters:
      :   `min` - the starting point
      :   `increment` - the increment
      :   `iterations` - the number of repetitions

      Returns:
      :   a series of doubles from a min incrementing by a given increment
          for a number of iterations


    - #### implementsInterface

      ```
      public static java.lang.Boolean implementsInterface(java.lang.Object object,
                                          java.lang.Class interf)
      ```


    - #### main

      ```
      public static void main(java.lang.String[] argv)
                       throws java.lang.Exception
      ```

      Main method for testing

      Parameters:
      :   `argv` -

      Throws:
      :   `java.lang.Exception`


    - #### readLocalProperties

      ```
      public static java.util.Properties readLocalProperties(java.lang.String resourceName)
                                                      throws java.lang.Exception
      ```

      Reads properties that inherit from three locations. Code from Weka:
      http://www.cs.waikato.ac.nz/ml/weka/
      Ian H. Witten and Eibe Frank (2005)
      "Data Mining: Practical machine learning tools and techniques", 2nd
      Edition, Morgan Kaufmann, San Francisco, 2005.
      Reads properties that inherit from three locations. Properties are first
      defined in the system resource location (i.e. in the CLASSPATH). These
      default properties must exist. Properties defined in the users home
      directory (optional) override default settings. Properties defined in the
      current directory (optional) override all these settings.

      Parameters:
      :   `resourceName` - the location of the resource that should be loaded. e.g.:
          "weka/core/Utils.props". (The use of hardcoded forward slashes
          here is OK - see jdk1.1/docs/guide/misc/resources.html) This
          routine will also look for the file (in this case)
          "Utils.props" in the users home directory and the current
          directory.

      Returns:
      :   the Properties

      Throws:
      :   `java.lang.Exception` - if no default properties are defined, or if an error
          occurs reading the properties files.


    - #### readProperties

      ```
      public static java.util.Properties readProperties(java.lang.String propertiesFilePath)
                                                 throws java.lang.Exception
      ```

      Reads properties Code adapted from Weka:
      http://www.cs.waikato.ac.nz/ml/weka/
      Ian H. Witten and Eibe Frank (2005)
      "Data Mining: Practical machine learning tools and techniques", 2nd
      Edition, Morgan Kaufmann, San Francisco, 2005.

      Returns:
      :   the Properties

      Throws:
      :   `java.lang.Exception` - if no default properties are defined, or if an error
          occurs reading the properties files.


    - #### roundDouble

      ```
      public static double roundDouble(double targetDouble,
                       int decimalPlace)
      ```

      Rounds a number to just n decimal digits

      Parameters:
      :   `targetDouble` - the number to be rounded
      :   `decimalPlace` - the number of digits to be left right of the decimal dot

      Returns:
      :   the rounded number


    - #### setFromObjectsToString

      ```
      public static java.util.TreeSet<java.lang.String> setFromObjectsToString(java.util.TreeSet<java.lang.Object> set)
      ```


- Overview
- Package
- Class
- Use
- Tree
- Deprecated
- Index
- Help

- Prev Class
- Next Class

- Frames
- No Frames

- All Classes

- Summary:
- Nested |
- Field |
- Constr |
- Method

- Detail:
- Field |
- Constr |
- Method
